# Severe Human Bocavirus–Associated Pneumonia in Adults at a Referral Hospital, Seoul, South Korea

**DOI:** 10.3201/eid2701.202061

**Published:** 2021-01

**Authors:** Sang-Ho Choi, Jin Won Huh, Sang-Bum Hong, Jiwon Jung, Min Jae Kim, Yong Pil Chong, Sung-Han Kim, Heungsup Sung, Eun Jin Chae, Kyung-Hyun Do, Sang-Oh Lee, Chae-Man Lim, Yang Soo Kim, Jun Hee Woo, Younsuck Koh

**Affiliations:** Asan Medical Center, University of Ulsan College of Medicine, Seoul, South Korea

**Keywords:** pneumonia, bocavirus, intensive-care units, co-infection, mortality, viruses, respiratory infections, HBoV, Seoul, South Korea

## Abstract

We report a case series of severe human bocavirus–associated pneumonia in adults in Seoul, South Korea. The virus accounted for 0.5% of all severe pneumonia cases. Structural lung disease and hematologic malignancy were common underlying diseases. Overall death rate was 54.5%. Higher death rates were associated with co-infection (83.3%) and immunocompromise (80.0%).

Human bocavirus (HBoV), a DNA virus in the *Parvoviridae* family, was first identified in 2005 (*1*). HBoV is distributed worldwide and has been found in 2%–33% of respiratory specimens, primarily from children with acute respiratory tract infection (*2*,*3*). In adults, HBoV is an uncommon cause of upper respiratory tract infection and pneumonia (*4*–*6*). It can also be associated with the acute exacerbation of chronic obstructive pulmonary disease (*7*). Recently, a few case reports have shown that HBoV can be associated with life-threatening pneumonia (*8*–*12*). However, severe HBoV-associated pneumonia has not been reported in a case series, and little is known about the characteristics of HBoV-associated pneumonia in critically ill adult patients. We investigated the incidence, clinical characteristics, and outcomes of severe HBoV-associated pneumonia in adults in Seoul, South Korea.

## The Study

We conducted a prospective observational cohort study of severe pneumonia in adult patients admitted to the medical intensive-care unit (ICU) at a 2,700-bed referral hospital in Seoul, South Korea, during March 2010–February 2019 (*13*,*14*). We initially included all adult patients admitted to the ICU with a diagnosis of pneumonia but later excluded patients with nonsevere pneumonia. We collected data on demographics, underlying diseases or conditions, immune status, seasonality, clinical manifestations, laboratory findings, pathogens, complications, treatment, and outcomes. This data collection was a routine part of the management and care of these patients at our hospital. Definitions and microbial evaluations are summarized in the Appendix. The Institutional Review Board of Asan Medical Center approved this study (approval no. 2010-0079) and waived informed-consent requirements.

During the study, 2,519 adult patients were admitted to the ICU with the diagnosis of severe pneumonia. After excluding 298 patients (83 community-acquired pneumonia [CAP] cases and 215 hospital-acquired pneumonia [HAP] cases) for whom multiplex respiratory virus PCR was not performed, 2,221 severe pneumonia patients (1,482 CAP cases and 739 HAP cases) were included. Among these 2,221 severe pneumonia patients, septic shock occurred in 1,306 (58.8%), and 2,141 (96.4%) required mechanical ventilation. Septic shock occurred in 80 patients (3.6%) who did not require mechanical ventilation.

Mean patient age was 65.8 years (range 16–97 years). Structural lung disease (26.9%) was the most common underlying disease in patients with CAP, and hematologic malignancy (25.4%) was the most common in patients with HAP (Appendix Table 1). One or more respiratory pathogens were identified in 1,510 patients (68.0%) (Appendix Table 2). Overall, 888 (40.0%) patients had bacterial infections, 711 (32.0%) had viral infections, and 230 (10.4%) patients had bacterial–viral co-infection. A total of 787 viruses were identified in 711 patients. Two viruses were identified in 60 patients and 3 viruses in 8 patients. Influenza virus (8.6%) and rhinovirus (8.4%) were the most common viral pathogens for severe CAP, whereas parainfluenza virus and respiratory syncytial virus were the most common viral pathogens for severe HAP.

Eleven HBoV-associated severe pneumonia cases (0.5% [11/2,221]) were reported. Of those, HBoV accounted for 0.4% (6/1,482) of CAP cases and 0.7% (5/739) of HAP cases. Of the 711 virus-associated severe pneumonia cases, HBoV accounted for 1.2% (6/501) of CAP cases and 2.4% (5/210) of HAP cases. Appendix Table 3 summarizes the characteristics and outcomes of the 11 patients with HBoV-associated severe pneumonia, which included 6 patients with CAP and 5 patients with HAP. HBoV occurred in all 4 seasons but was more common during September–February (8 cases). Nine patients were men (81.8%); the median age was 69.0 years (range 36–81 years). All patients had >1 severe underlying diseases. Structural lung disease (5 patients) and hematologic malignancy (4 patients) were the most common underlying illnesses. Five patients (45.5%) were immunocompromised.

Viruses were detected by using nasopharyngeal aspirate or swab specimens. In 1 patient, the virus was detected in bronchoalveolar lavage fluid and nasopharyngeal samples. Co-infection was observed in 6 patients (54.5%). Eight patients underwent chest computed tomography. The most common radiologic findings were bilateral and multifocal consolidation and ground-glass opacity.

The median length of ICU stay was 9.0 days (range 1–74 days). Overall death rate was 54.5% (6/11). The death rate was 80.0% (4/5) for immunocompromised patients and 33.3% (2/6) for immunocompetent patients. Higher death rates were observed in cases of co-infection (83.3%, 5/6) than in cases of sole HBoV infection (20.0% [1/5]) (p = 0.08). All immunocompromised patients with co-infection died (3/3), whereas no immunocompetent patients without co-infection died (0/3).

## Conclusions

Our study demonstrated that HBoV is an uncommon pathogen for adult patients with severe pneumonia requiring ICU admission. All episodes we investigated occurred in patients with serious underlying diseases, and co-infection was frequent. Overall death rates were high and closely associated with immunocompromised state and presence of co-infection.

Information on severe HBoV-associated pneumonia in adults is limited. Five cases of severe HBoV-associated pneumonia have been reported to date (*8*–*12*), which included 3 cases from the same facility in Germany (*9*,*10*,*12*). Of the 5 patients, 3 had hematologic malignancy and 1 had cystic fibrosis. One of the patients was a 74-year-old immunocompetent man (*10*). He had an acute head injury and rib fracture, probably because of weakness from HBoV pneumonia, necessitating mechanical ventilation. In our study, patients with structural lung diseases, including chronic obstructive pulmonary disease and bronchiectasis, were predisposed to severe HBoV-associated pneumonia. Most of these patients were not immunocompromised and had CAP. Therefore, clinicians should consider HBoV as an uncommon pathogen of severe pneumonia in adults with structural lung disease.

Consistent with previous reports (*4*,*5*), we found a high rate of co-infection with other pathogens in patients with HBoV infection. HBoV has shown a prolonged persistence in the mucosa of the respiratory tract. Viral persistence contributes to the high frequency of coinfections with proper respiratory pathogens (*15*). This phenomenon might be associated with the underlying severe diseases. Of note, co-infection was closely related to higher overall death rates in our patients. Our series included 5 cases of sole HBoV infection, which was more common in CAP patients and associated with lower death rates. These findings indicated that HBoV itself has a lower virulence potential and rarely causes severe pneumonia, which is predominant in immunocompromised patients or patients with underlying structural lung disease. The higher incidence of severe HBoV-associated pneumonia in HAP patients compared with CAP patients (0.7% vs. 0.4%) might be explained by the higher proportion of immunocompromised patients in the HAP population.

Our study has some limitations. First, we excluded 298 patients (83 of 1,565 CAP patients [5.8%] and 215 of 954 HAP patients [22.5%]) for whom multiplex respiratory virus PCR was not performed. Because our study was observational, microbial evaluations and patient-management decisions were made by attending physicians, and multiplex respiratory virus PCR test was not used for all patients. Therefore, selection bias might have occurred, especially for HAP. Second, HBoV was mostly identified through nasopharyngeal specimens only and was frequently accompanied by copathogens. Therefore, we could not evaluate in detail the virulence potential of sole HBoV infection. Finally, we did not conduct a genotypic study of HBoV, and the viral load therefore was not tested.

In summary, in this study, HBoV accounted for 0.5% of severe pneumonia cases in adults. HBoV-associated severe pneumonia could lead to high death rates. Underlying severe diseases and frequent co-infection seem to be responsible for poor outcomes.

Appendix Additional information about severe human bocavirus–associated pneumonia in adults at a referral hospital, Seoul, South Korea.
