# Supplementary material for: Severe Human Bocavirus–Associated Pneumonia in Adults at a Referral Hospital, Seoul, South Korea
Source: Emerg Infect Dis. 2021 Jan;27(1):226–8. doi: 10.3201/eid2701.202061 (PMC7774574; doi:10.3201/eid2701.202061)
Supplement: Appendix — Additional information about severe human bocavirus–associated pneumonia in adults at a referral hospital, Seoul, South Korea. [file 20-2061-Techapp-s1.pdf]

Article DOI: https://doi.org/10.3201/eid2701.202061

Severe Human Bocavirus–Associated Pneumonia in Adults at a Referral Hospital, Seoul, South Korea

Appendix

Definitions

Pneumonia was defined as the presence of a new infiltration on a chest radiograph plus ≥1 of the following: fever (temperature ≥38°C) or hypothermia (temperature <35.0°C), new cough with or without sputum production, pleuritic chest pain, dyspnea, and altered breath sound on auscultation (*1*). Severe pneumonia was diagnosed when the patient required vasopressors for shock or mechanical ventilation for respiratory failure. The respiratory pathogens identified from specimens collected ≤72 hours after the diagnosis of pneumonia were considered pneumonia pathogens. Hospital-acquired pneumonia (HAP) was defined as pneumonia that occurred ≥48 hours after admission and did not appear to be incubating at the time of admission (*2*). Otherwise, the pneumonia was categorized as community-acquired pneumonia (CAP). An immunocompromised state was defined as one of the following conditions: (i) daily receipt of immunosuppressants, including corticosteroids; (ii) human immunodeficiency virus infection; (iii) receipt of solid organ or hematopoietic stem cell transplantation; (iv) receipt of chemotherapy for underlying malignancy in the previous 6 months; and (v) presence of underlying immune deficiency disorder (*3*).

Microbial Evaluations

The microbial evaluations were determined by the attending physicians, taking into consideration the patient's immune status, clinical course, acquisition site, and radiographic features. The microbial evaluations included the following: 1) bacteria: 3 sets of blood cultures; sputum or endotracheal aspirates, or BAL fluid Gram staining and culture; PCR for *Mycoplasma pneumoniae*, *Chlamydia pneumoniae*, and *Legionella pneumophila* using an AmpliSens *M.*

pneumoniae/*C. pneumoniae*-FRT PCR kit and AmpliSens *L. pneumophila*-FRT PCR kit (InterLabService Ltd, https://www.interlabservice.ru/en/); urinary antigen test using the BinaxNOW kit for *Streptococcus pneumoniae* and *L. pneumophila* serogroup 1 (Abbott, https://www.abbott.com/); 2) viruses: nasopharyngeal aspirates or BAL fluid multiplex reverse transcription PCR assay using the Anyplex II RV 16 Detection kit or Allplex Respiratory Panel 1, 2, 3 (Seegene Inc., http://www.seegene.com/); BAL fluid shell vial culture for influenza virus, respiratory syncytial virus, parainfluenza virus, adenovirus, and cytomegalovirus (Diagnostic Hybrids, Inc., https://www.quidel.com/); 3) mycobacteria: sputum or endotracheal aspirates, or BAL fluid Ziehl-Neelsen staining; combination of solid media culture (Ogawa medium, Korean Institute of Tuberculosis, Seoul, South Korea) and liquid media culture using a BACTEC 960 Mycobacterial Growth Indicator Tube (BD, https://www.bd.com/en-us); identification of *Mycobacterium tuberculosis* and nontuberculous mycobacteria (NTM) by PCR using AdvanSure TB/NTM real-time PCR (LG Chem Life Sciences, https://www.lgchem.com/main/index); NTM identification using GenoType mycobacterium CM/AS (Hain Lifescience GmbH, https://www.hain-lifescience.de/en/); 4) fungi: sputum or endotracheal aspirates, or BAL fluid fungus staining and culture; serum or BAL fluid *Aspergillus* galactomannan antigen assay using a Platelia *Aspergillus* Antigen Kit (Bio-Rad, https://www.bio-rad.com/); direct fluorescence assay using Light Diagnostics *Pneumocystis carinii* DFA Kit (Millipore Sigma, https://www.emdmillipore.com/US/en) or real-time PCR assay using an AmpliSens *Pneumocystis jirovecii* (carinii)-FRT PCR kit (InterLabService Ltd, https://www.interlabservice.ru/en/).

References

1. Carratalà J, Mykietiuk A, Fernández-Sabé N, Suárez C, Dorca J, Verdaguer R, et al. Health care-associated pneumonia requiring hospital admission: epidemiology, antibiotic therapy, and clinical outcomes. Arch Intern Med. 2007;167:1393–9. PubMed https://doi.org/10.1001/archinte.167.13.1393

2. American Thoracic SocietyInfectious Diseases Society of America. Guidelines for the management of adults with hospital-acquired, ventilator-associated, and healthcare-associated pneumonia. Am J Respir Crit Care Med. 2005;171:388–416. PubMed https://doi.org/10.1164/rccm.200405-644ST

3. Micek ST, Kollef KE, Reichley RM, Roubinian N, Kollef MH. Health care-associated pneumonia and community-acquired pneumonia: a single-center experience. Antimicrob Agents Chemother. 2007;51:3568–73. PubMed https://doi.org/10.1128/AAC.00851-07

Appendix Table 1. Demographics and underlying diseases/conditions of 2,221 patients with severe pneumonia*

	No. (%)		
Identified virus(es)	Total (n = 2,221)	CAP† (n = 1,482)	HAP (n = 739)
Demographics			
Male sex	1,583 (71.3)	1,056 (71.3)	527 (71.3)
Median age (range), year	68.0 (16–97)	69.0 (16–97)	65.0 (16–97)
Underlying diseases or conditions‡			
Diabetes mellitus	551 (24.8)	378 (25.5)	173 (23.4)
Structural lung disease	542 (24.4)	398 (26.9)	144 (19.5)
Chronic obstructive pulmonary disease	254 (11.4)	190 (12.8)	64 (8.7)
Interstitial lung disease	165 (7.4)	118 (8.0)	47 (6.4)
Tuberculosis-destroyed lung	61 (2.7)	42 (2.8)	19 (2.6)
Bronchiectasis	63 (2.8)	48 (3.2)	15 (2.0)
Pneumoconiosis	9 (0.4)	7 (0.5)	2 (0.3)
Bronchiolitis obliterans	7 (0.3)	4 (0.3)	3 (0.4)
Solid cancer	484 (21.8)	317 (21.4)	167 (22.6)
Hematologic malignancy	337 (15.2)	149 (10.1)	188 (25.4)
Congestive heart failure	101 (4.5)	69 (4.7)	32 (4.3)
Chronic renal failure	91 (4.1)	67 (4.5)	24 (3.2)
End-stage renal disease	90 (4.1)	58 (3.9)	32 (4.3)
Immunocompromised state	1,029 (46.3)	632 (42.6)	397 (53.7)

*Data are presented as the number (percentage) of patients. CAP, community-acquired pneumonia; HAP, hospital-acquired pneumonia.
†Included 946 patients with healthcare-associated community-onset pneumonia.
‡Some patients had ≥1 underlying diseases or conditions.

Appendix Table 2. Identity of pathogens in 2,221 adult patients with severe pneumonia*

	No. (%)		
Identified organism	Total (n = 2,221)	CAP† (n = 1,482)	HAP (n = 739)
None	711 (32.0)	483 (32.6)	228 (30.9)
Bacteria	888 (40.0)	575 (38.8)	313 (42.4)
S. pneumoniae	126 (5.7)	114 (7.7)	12 (1.6)
S. aureus	197 (8.9)	112 (7.6)	85 (11.5)
Methicillin-susceptible	58 (2.6)	51 (3.4)	7 (0.9)
Methicillin-resistant	139 (6.3)	61(4.1)	78 (10.6)
L. pneumophila	34 (1.5)	30 (2.0)	4 (0.5)
H. influenzae	14 (0.6)	13 (0.9)	1 (0.1)
M. catarrhalis	13 (0.6)	12 (0.8)	1 (0.1)
C. striatum	13 (0.6)	3 (0.2)	10 (1.4)
S. pyogenes	6 (0.3)	5 (0.3)	1 (0.1)
Nocardia species	3 (0.1)	3 (0.2)	0
S. constellatus	3 (0.1)	2 (0.1)	1 (0.1)
Group G streptococcus	2 (0.1)	2 (0.1)	0
S. agalactiae	2 (0.1)	2 (0.1)	0
S. anginosus	1 (0)	0	1 (0.1)
R. mucilaginosa	1 (0)	1 (0.1)	0
Enteric Gram-negative bacilli	270 (12.2)	196 (13.2)	74 (10.0)
K. pneumoniae	166 (7.5)	119 (8.0)	47 (6.4)
E. coli	64 (2.9)	50 (3.4)	14 (1.9)
E. cloacae	16 (0.7)	11 (0.7)	5 (0.7)
K. aerogenes	10 (0.5)	8 (0.5)	2 (0.3)
S. marcescens	8 (0.4)	7 (0.5)	1 (0.1)
C. freundii	6 (0.3)	3 (0.2)	3 (0.4)
K. oxytoca	3 (0.1)	1 (0.1)	2 (0.3)
K. ozaenae	2 (0.1)	2 (0.1)	0
M. morganii	2 (0.1)	2 (0.1)	0
P. mirabilis	2 (0.1)	1 (0.1)	1 (0.1)
P. stuartii	2 (0.1)	2 (0.1)	0
Non-enteric Gram-negative bacilli	284 (12.8)	135 (9.1)	149 (20.2)
P. aeruginosa	126 (5.7)	86 (5.8)	40 (5.4)
A. baumannii	125 (5.6)	41 (2.8)	84 (11.4)
S. maltophilia	29 (1.3)	5 (0.3)	24 (3.2)
A. xylosoxidans	5 (0.2)	3 (0.2)	2 (0.3)
P. fluorescens	1 (0.1)	1 (0.1)	1 (0.1)

Identified organism	No. (%)		
	Total (n = 2,221)	CAP† (n = 1,482)	HAP (n = 739)
A. lwoffii	1 (0.1)	1 (0.1)	1 (0.1)
C. indologenes	1 (0)	0	1 (0.1)
Atypical pathogen	24 (1.1)	23 (1.6)	1 (0.1)
M. pneumoniae	11 (0.5)	11 (0.7)	0
O. tsutsugamushi	9 (0.4)	9 (0.6)	0
C. pneumoniae	6 (0.3)	5 (0.3)	1 (0.1)
Virus	711 (32.0)	501 (33.8)	210 (28.4)
Rhinovirus	165 (7.4)	125 (8.4)	40 (5.4)
Influenza virus	165 (7.4)	127 (8.6)	38 (5.1)
Influenza A	133 (6.0)	103 (7.0)	30 (4.1)
Influenza B	32 (1.4)	24 (1.6)	8 (1.1)
Parainfluenza virus	121 (5.4)	71 (4.8)	50 (6.8)
Type 3	73 (3.3)	33 (2.2)	40 (5.4)
Type 1	24 (1.1)	18 (1.2)	6 (0.8)
Type 4	20 (0.9)	15 (1.0)	5 (0.7)
Type 2	5 (0.2)	5 (0.3)	0
Respiratory syncytial virus	100 (4.5)	54 (3.6)	46 (6.2)
Respiratory syncytial virus A	55 (2.5)	28 (1.9)	27 (3.7)
Respiratory syncytial virus B	46 (2.1)	27 (1.8)	19 (2.6)
Human coronavirus	85 (3.8)	64 (4.3)	21 (2.8)
229E/NL63	43 (1.9)	33 (2.2)	10 (1.4)
OC43/HKU1	43 (1.9)	31 (2.1)	12 (1.6)
Human metapneumovirus	57 (2.6)	50 (3.4)	7 (0.9)
Cytomegalovirus	34 (1.5)	20 (1.3)	14 (1.9)
Adenovirus	27 (1.2)	15 (1.0)	12 (1.6)
Herpes simplex virus 1	14 (0.6)	9 (0.6)	5 (0.7)
Bocavirus	11 (0.5)	6 (0.4)	5 (0.7)
Enterovirus	6 (0.3)	4 (0.3)	2 (0.3)
Mycobacterium	52 (2.3)	46 (3.1)	6 (0.8)
M. tuberculosis	39 (1.8)	34 (2.3)	5 (0.7)
Non-tuberculous mycobacterium	13 (0.6)	12 (0.8)	1 (0.1)
M. intracellulare	7 (0.3)	6 (0.4)	1 (0.1)
M. avium	1 (0)	1 (0.1)	0
M. peregrinum	1 (0)	1 (0.1)	0
Unspecified	3 (0.1)	3 (0.2)	0
Fungus	210 (9.5)	123 (8.3)	87 (11.8)
Aspergillus species	128 (5.8)	66 (4.5)	62 (8.4)
Pneumocystis jirovecii	83 (3.7)	58 (3.9)	25 (3.4)
Rhizopus species	3 (0.1)	1 (0.1)	2 (0.3)
Cunninghamella species	3 (0.1)	1 (0.1)	2 (0.3)
Trichosporon asahii	2 (0.1)	1 (0.1)	1 (0.1)
Candida tropicalis	1 (0)	0	1 (0.1)
Penicillium species	1 (0)	1 (0.1)	0

*Data are presented as the number (percentage) of patients. CAP, community-acquired pneumonia; HAP, hospital-acquired pneumonia.
†Included 946 patients with healthcare-associated community-onset pneumonia.

Appendix Table 3. Characteristics of 11 patients with severe bocavirus-associated pneumonia admitted between March 2010 and February 2019*

Patient no.	Category of pneumonia	Year/month	Age/sex	Underlying disease or condition	Immunocompromised state†	PCR-positive specimen	Copathogen‡	CT findings	Outcome (cause of death)
1	CAP	2014/Feb	79/male	COPD, heart failure	No	NP, BAL fluid	None	Diffuse and bilateral GGO with consolidation	Alive
2	CAP	2015/Jan	63/male	Diabetes mellitus, cerebrovascular attack	No	NP	*E. coli*	Multifocal patchy GGO with consolidation	Died on hospital day 19 (central-line associated *A. baumannii* bacteremia)
3	CAP	2015/Apr	75/male	Bronchiectasis	No	NP	None	Multifocal patchy consolidation	Alive
4	CAP	2015/Sep	74/male	COPD	No	NP	None	Consolidation and bronchial wall thickening on right upper lobe	Alive
5	CAP	2015/Nov	69/male	Ischemic heart failure	No	NP	*M. pneumoniae*	Diffuse and bilateral GGO with consolidation	Alive
6	CAP	2015/Dec	80/male	Idiopathic pulmonary fibrosis on steroids	Yes	NP	None	Multifocal patchy GGO with consolidation	Died on hospital day 44 (*P. aeruginosa* ventilator-associated pneumonia)
7	HAP	2011/May	54/female	Acute lymphocytic leukemia	Yes	NP	*A. baumannii* + parainfluenza virus type 3	Chest x-ray: Multifocal patchy consolidation and increased interstitial marking in both lungs	Died on ICU day 2 (refractory shock)
8	HAP	2011/Jun	63/female	Primary central nervous system lymphoma	Yes	NP	None	Multifocal patchy consolidation and interstitial thickening	Alive
9	HAP	2012/Sep	51/male	Acute myeloid leukemia	Yes	NP	Parainfluenza virus type 4	Chest x-ray: Ill-defined consolidation and GGO in left lower lung zone	Died on ICU day 10 (refractory shock)
10	HAP	2017/Nov	81/male	Tuberculosis-destroyed lung	No	NP	MRSA + rhinovirus	Chest x-ray: multifocal patchy GGO and consolidation	Died on ICU day 72 (intracranial hemorrhage)
11	HAP	2019/Jan	36/male	Acute lymphoid leukemia	Yes	NP	Influenza A	Chest x-ray: Multifocal patchy consolidation and increased interstitial marking in both lungs	Died on ICU day 9

*BAL, bronchoalveolar lavage; CAP, community-acquired pneumonia; COPD, chronic obstructive pulmonary disease; CT, computed tomography; GGO, ground-glass opacity; HAP, hospital-acquired pneumonia; ICU, intensive care unit; MRSA, methicillin-resistant *Staphylococcus aureus;* NP, nasopharyngeal swab or aspirate; PCR, polymerase chain reaction.
†Defined as one of the following conditions: (i) daily receipt of immunosuppressants, including corticosteroids; (ii) human immunodeficiency virus infection; (iii) receipt of solid organ or hematopoietic stem cell transplantation; (iv) receipt of chemotherapy for underlying malignancy in the previous 6 mo; and (v) presence of underlying immune deficiency disorder.
‡Respiratory pathogen(s) identified from specimens collected $\leq$72 hours after diagnosis of pneumonia.
